# Permanent draft genome sequence of *Bradyrhizobium vignae,* strain ISRA 400, an elite nitrogen-fixing bacterium, isolated from the groundnut growing area in Senegal

**DOI:** 10.7150/jgen.88302

**Published:** 2023-11-01

**Authors:** Diariatou NIANG, Abdellatif GUEDDOU, Nogaye NIANG, Darius NZEPANG, Aissatou SAMBOU, Adama DIOUF, Arlette Z ZAIYA, Maimouna CISSOKO, Djamel GULLY, Joel-Romaric NGUEPJOP, Sergio SVISTOONOFF, Daniel FONCEKA, Valérie HOCHER, Diégane DIOUF, Saliou FALL, Louis S. TISA

**Affiliations:** 1 Université Cheikh Anta Diop (UCAD/FST), Département de Biologie Végétale, École doctorale Sciences de la Vie, de la Santé et de l'Environnement (EDSEV), B.P.: 5005 Dakar-Fann, Senegal.; 2 Institut Sénégalais de Recherche Agricole (ISRA), Laboratoire National de Recherches sur la Productions Végétales (LNRPV), Campus ISRA-IRD de Bel air, Dakar.; 3 Laboratoire Commun de Microbiologie (LCM: IRD - ISRA - UCAD), B. P. 3120, Campus ISRA-IRD de Bel air, Dakar.; 4 Department of Molecular, Cellular, and Biomedical Sciences, University of New Hampshire, Durham, New Hampshire, USA.; 5 Institut Sénégalais de Recherche Agricole (ISRA), Centre d'Etudes Régional pour l'Amélioration de l'Adaptation à la Sécheresse, CERAAS - Route de Khombole, BP3320 Thiès, Senegal.; 6 Institut de Recherche pour le Développement (IRD), UMR PHIM IRD/INRAE/CIRAD/U.Montpellier/Institut Agro , Montpellier, France.; 7 CIRAD, UMRAGAP, CIRAD/Univ Montpellier/ INRAE, Institut Agro, F-34398 Montpellier, France.; 8 Université du Sine Saloum El Hadj Ibrahima Niass (USSEIN), UFR Sciences sociales et environnementales, Centre d'Excellence Africain "Agriculture pour la Sécurité Alimentaire et Nutritionnelle" (CEA-AGRISAN), Kaolack.; 9 Present address: Institute of Agricultural Research for Development (IRAD), Yaounde, Cameroon.

**Keywords:** *Bradyrhizobium*, * Arachis hypogaea*, biological nitrogen fixation, genome, symbiosis

## Abstract

A new *Bradyrhizobium vignae* strain called ISRA400 was isolated from groundnut (*Arachis hypogaea* L.) root nodules obtained by trapping the bacteria from soil samples collected in the Senegalese groundnut basin. In this study, we present the draft genome sequence of this strain ISRA400, which spans approximatively 7.9 Mbp and exhibits a G+C content of 63.4%. The genome analysis revealed the presence of 48 tRNA genes and one rRNA operon (16S, 23S, and 5S). The nodulation test revealed that this strain ISRA400 significantly improves the nodulation parameters and chlorophyll content of the *Arachis hypogaea* variety Fleur11. These findings suggest the potential of *Bradyrhizobium vignae* strain ISRA400 as an effective symbiotic partner for improving the growth and productivity of groundnut crop.

## Introduction

Rhizobia are soil bacteria that establish a symbiotic nitrogen-fixing association with legumes, thereby improving the nutritional status and fitness of the host plant 1; 2. This mutualistic association leads to the formation of a root nodule structure, where Biological Nitrogen Fixation (BNF) takes place. BNF is a beneficial process for legumes as it provides biologically available nitrogen to the plant, which is a crucial component limiting plant growth 3; 4. Considering each year, BNF contributes approximately 40 million tons of combined nitrogen worldwide 5, the essential role played by efficient rhizobia in maintaining sustainable agriculture becomes evident, especially for cash crops such as groundnut (*Arachis hypogaea* L.) 5. Groundnut is one of the most important oilseed legumes worldwide forming effective root nodules primarily with slow-growing bacteria belonging to the genus *Bradyrhizobium* 5; 6; 7. Phylogenetic analyses of Bradyrhizobium indicate that it has three main lineages, commonly referred to as supergroups or superclades 8; 9; 10. These supergroups are represented by B. japonicum, B. elkanii and the photosynthetic Bradyrhizobium species 8; 11; 10. Currently, the genus *Bradyrhizobium* consists of 41 assigned species 12. Recent development of next-generation sequencing (NGS) has facilitated the acquisition of genome-wide sequence data, leading to the availability of whole genomic sequences for many strains of several bacterial species, including Bradyrhizobia 16. The National Center for Biotechnology Information Database reports the sequencing of 37 *Bradyrhizobium* strains isolated from different species, including soybean, *Aeschynomene*, and wheat 13.

In Senegal, groundnut plays an important role in production systems and contributes over 40% of the rural cash income for small family farms due to its multiple uses in food and fodder (seed, oil, cake, husks and shells) 14. In a previous study, a collection of 35 symbiotic *Bradyrhizobia* isolated from root nodules of groundnut plants sampled in different agroecological areas of the Senegalese groundnut basin were characterized using multilocus sequence analysis (MLSA) of the 16s/23s intergenic region (IGS) and the nodC gene 15. Surprisingly, these strains were classified into three clusters which exhibit contrasted symbiotic characteristics on groundnut 15. Among them, the strain referred to as ISRA400, potentially related to the already described species *Bradyrhizobium vignae* 15 has shown its agronomic relevance and symbiotic properties and is thus an interesting strain to study at the genomic level. In this study, we report on the sequencing results of the genome of the strain ISRA400, a symbiont of *Arachis hypogaea* L., isolated from the groundnut basin of Senegal. These genomic data will prove valuable in identifying genes associated with symbiotic performance and host compatibility on several legume species.

### Isolation of the *Bradyrhizobium vignae* strain ISRA400

**Figure 1:**
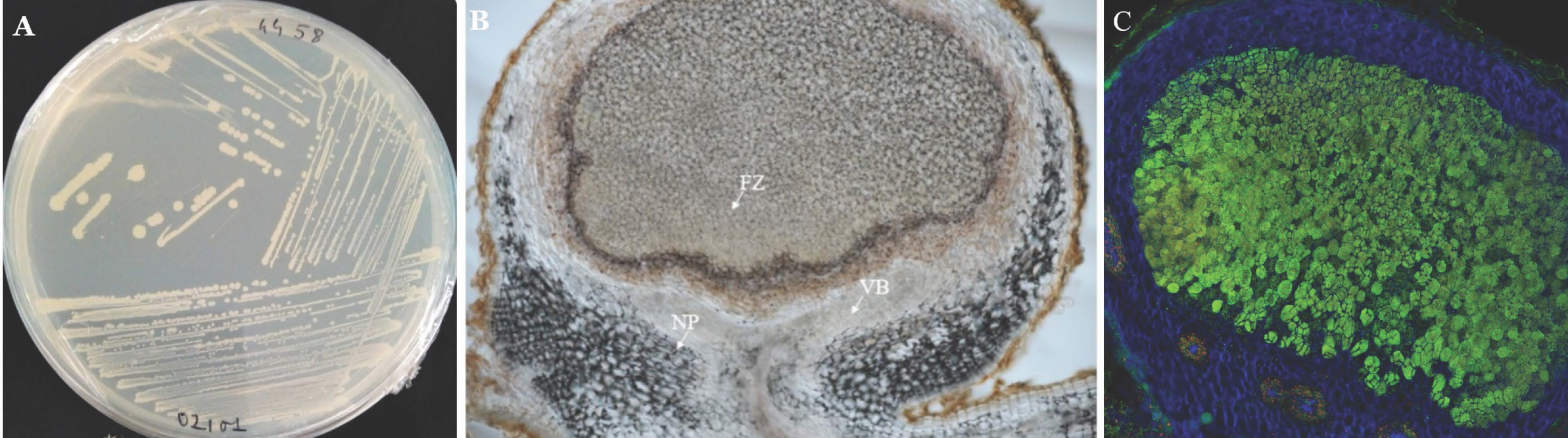
** Observation of *Bradyrhizobium vignae* strain ISRA400 in pure culture and in groundnut nodule. A**: Colonies of *B. vignae* strain ISRA400 incubated on YEMA solid medium. **B**: Longitudinal section of a 35-day post inoculation (dpi) nodule obtained after inoculation of *A. hypogeae* seedlings of the variety Fleur11 with *B. vignae* ISRA400. **C**: Nodule section observed under confocal microscopy showing live (green) rhizobia in the fixation zone, surrounded by plant cells (blue). Scale bar = 100µm. FZ: fixation zone, NP: nodule parenchyma; VB: Vascular bundles.

Isolation of strain ISRA400 was performed on groundnut root nodules obtained through plant trapping the bacteria from a soil sample collected in the Senegalese groundnut basin. The nodules were subjected to the following steps: immersion in 70% (v/v) ethanol for 2 minutes, incubation in 3% calcium hypochlorite for 3 minutes, followed by six washes with sterile water. Subsequently, the nodules were ground in a 2 ml tube containing sterile water. The resulting crushed nodules were spread onto Petri dishes containing Yeast Extract-Mannitol Agar solid culture medium (YEMA) 17. The plates were incubated at 28°C for 7 days 17. To ensure a pure culture, ISRA400 strain was streaked to produce a single colony three times (Figure 1) and selected for its ability to nodulate the host plant (*A. hypogaea* L.,) under controlled conditions.

### Sequencing of the *Bradyrhizobium vignae* strain ISRA400

High quality gDNA of *B. vignae* strain ISRA400 was extracted using CTAB method 18. Sequencing of the draft genome of *B. vignae* strain ISRA400 was performed at the Hubbard Center for Genome Studies (University of New Hampshire, Durham, NH) using Illumina HiSeq2500 platform technology A standard Illumina shotgun library was constructed and sequenced using the Illumina HiSeq2500 platform generating. The pair-end library generated 13,461,402 reads of 250 bp in length. The Illumina sequence data were trimmed and assembled by CLC Genomic Workstation software (v. 21.0.1) The final draft assembly for *B. vignae strain* ISRA400 consisted of 157 contigs with an N_50_ contig size of 87,613 and 420.0x coverage of the genome.

**Figure 2:**
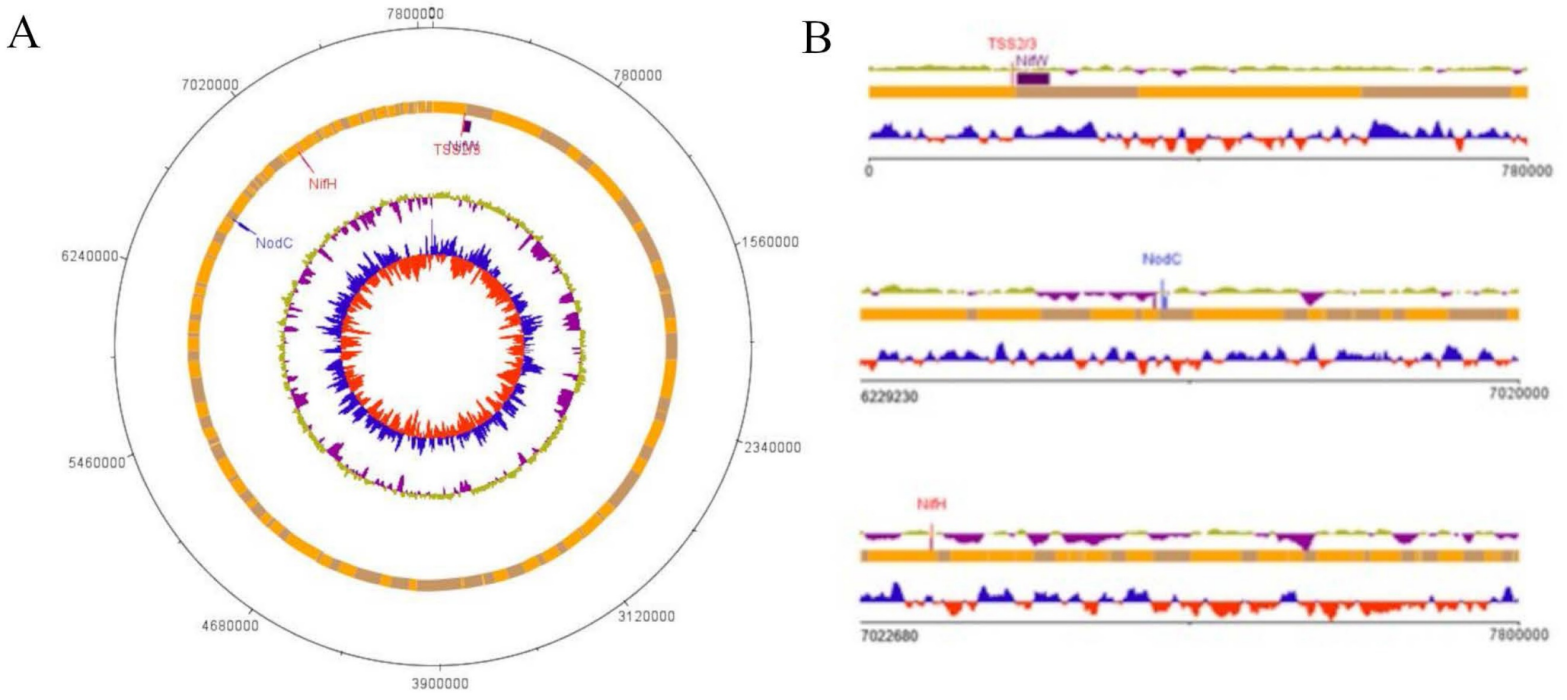
** Schematic representations of the *B. vignae* ISRA400 chromosome. A**: Circular representation of the genome. The bars in the outermost circle show the positions of the genes in a clockwise direction. The bars in the second circle represent the regions corresponding to genes involved in nodulation and nitrogen fixation (Symbiosis Island). The third circle shows the average GC percentage (yellow and purple) and the innermost circle shows the GC skewness values (blue and red) B**:** Linear representation of parts of the chromosome focusing on symbiotic genes. This map was plotted using the DNAplotter program 24.

The final assembled genome contained a total sequence length of 7,857,394 bp with a G+C content of 63.4 %, which is within the range of members of the genus *Bradyrhizobium* 19. The assembled *B. vignae strain* ISRA400 genome was annotated *via* the NCBI Prokaryote Genome Annotation Pipeline (PGAP) v4.13 20 and resulted in 7,345 candidate protein-encoding genes, 48 tRNA genes and tree rRNA genes identified only 1 operon (16S, 23S and 5S). No plasmids were detected, which is a common feature of genomes in this genus 21. The genome contains a large symbiotic island that contains most of the key nitrogen fixation related genes including *nifH, nifA, nifv, nifw, nodBCDI.* This region was detected at position 6597228-7106102 bp of the genome and contained about 508 kb of the chromosome (Figure 2). The *nodBCDI* genes encode a set of proteins crucial for the initial stages of nodulation, while the *nif* genes code for the enzyme nitrogenase. The *nod* genes are responsible for synthesizing lipo-chitooligosaccharides, also known as Nod factors, in response to plant molecular signals, primarily flavonoids. These Nod factors induce significant cell division in the root cortex and trigger other changes that ultimately lead to nodule formation 22. Furthermore, the genome of *Bradyrhizobium vignae* strain ISRA400 is equipped with type II and III protein secretory systems (located at coordinates 170,312 - 171,826). The type III secretory system (T3SS) is a complex secretory apparatus that enables the direct injection of proteins (called effectors) into the cytoplasm of the host plant cell 2. These effectors may also circumvent the need for nodulation (Nod) factors to trigger nodule formation 23. The genomic sequence data reported in this study could thus be useful in identifying effectors governing the establishment of the T3SS-dependent symbiotic process in cultivated legumes. This could help identifying genes essential for host compatibility and optimal performance of *B. vignae* strain ISRA400 in effective interaction with various tropical legumes important for agriculture.

A whole genome-based taxonomic analysis *via* the Type (Strain) Genome Server (TYGS) platform 25 https://tygs.dsmz.de/) including digital DNA: DNA hybridization (dDDH) values was performed together with a comparison of the G+C content. The results confirm that strain ISRA400 is closely related to the already described species *B. vignae* type strain 7-2 ^T^ which was isolated from an effective nodule of *Vigna unguiculata* in the Kavango region of Namibia 26. These results confirm that *Bradyrhizobium vignae* is a species with diverse symbiovars.

### Efficacy of *B. vignae* ISRA400 on groundnut nodulation

The symbiotic efficacy of the *B. vignae* ISRA400 strain was assessed on the Fleur11 groundnut variety, in a culture room (photoperiod: 16h day and 8h night, light intensity: 2500- 4000 Lux, temperature: 28°C). Fleur11 is an improved groundnut Spanish type cultivar known for its high yield and short growth cycle of approximately 90 days. This cultivar is widely grown in the Senegalese groundnut basin and has been utilized as one of the parental lines in various interspecific (cultivated x wild) populations 14.

Two treatments were compared under a randomized block design with four replicates. The treatments were as follows: 1 two-day-old seedlings of *A. hypogaea* variety Fleur 11 were inoculated with *Bradyrhizobium Vignae* ISRA400 and 2 uninoculated plantlets where no *Bradyrhizobium vignae* ISRA400 was added served as a control (NI).

*Bradyrhizobium Vignae* ISRA400 was cultured in Erlenmeyer flasks containing 50 mL of liquid Yeast Extract-Mannitol culture medium (YEM). The flasks were incubated 72h on an orbital shaker at 200 rpm at 28 °C. After incubation, the bacterial cells were precipitated by centrifugation and further resuspended in a phosphate saline buffer prior to inoculation. Fleur 11 groundnut seeds were disinfected by immersing them in 70% alcohol for 30 seconds and followed by incubating in a 2% sodium hypochlorite solution for 60 seconds. The seeds were rinsed five times with sterile distilled water and placed on Petri dishes containing agar water 0,8% w/v) for germination. Two-day-old seedlings were transferred in 11 cm diameter pots each containing 200g sterile vermiculite. For the inoculation process, 10 ml of the bacterial suspension (approximately 10^8^ bacterial cells) was used to inoculate the rootlets of groundnut seedlings.

After three weeks incubation in a culture room (photoperiod: 16h day and 8h night, light intensity: 2500- 4000 Lux, temperature: 28°C), nodulation was observed. This time period was during the flowering stage when legumes typically have the highest number of active nodules 28. The nodules were carefully separated from the roots and the nodule number (NN) was recorded. Chlorophyll content was estimated with a chlorophyll meter SPAD-502 Plus (Konica Minolta). This device provides an indirect measurement of the chlorophyll content in leaves 27. It works by pinching the leaf tip, and the SPAD meter displays an estimated chlorophyll content value.

Data were statistically analyzed by using ANOVA, and the means were compared using Newman and Keuls test (p < 0.05%). The results are presented in Table 1.

**Figure 3:**
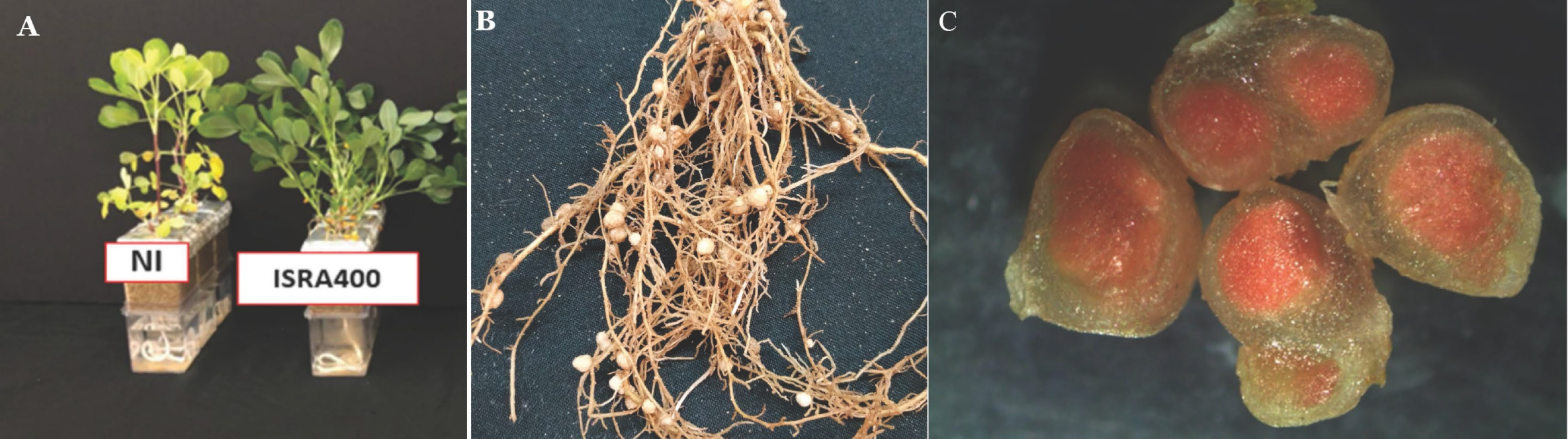
** Nodulation of *A. hypogaea* variety Fleur 11 inoculated with *B. vignae* ISRA400 strain. A**: Comparison of inoculated and non-inoculated plants. The inoculation with the *B. vignae* ISRA400 bacterial strain leds to a better plant development and health (green color). **B**: Plant root system with nodules. **C**: Freehand nodule sections showing the presence of leghemoglobin (red color) indicating that the nitrogen fixation is effective. Scale = 1mm

Previous research has already identified *B. vignae* ISRA400 as a promising candidate for the Fleur11 groundnut variety under both laboratory and greenhouse conditions (data not shown) 8. Here, we observed that at the flowering stage that the nodule was medium in size and red in color, indicating that N_2_-fixing activity wass higher due to active enzyme activity and proper bacterial cell growth (Figure 3) 28.

Based on the results obtained, it is evident that *B. vignae* ISRA400 exhibits a high level of effectiveness in contributing nitrogen to the peanut plants (Figure 3C). Therefore, it has the potential to be recommended as an inoculant for groundnut cultivation in Senegal, where Fleur11 is widely grown. The establishment of symbiotic associations between rhizobia and legumes plays a critical role in enhancing nitrogen availability in agricultural systems. Indeed, studying the diversity of symbiotic bacteria that nodulate groundnut is essential for the development of rhizobial inoculants that are well-adapted to the specific environmental conditions of local growing areas and could thus participate in reducing the reliance on synthetic fertilizers, often costly and having negative environmental impacts.

### Nucleotide sequence accession number

The draft genome sequence of the *Bradyrhizobium vignae* strain 'ISRA400' have been deposited DDBJ/EMBL/GenBank under the accession number JAGIKT000000000.Both the assembly and raw reads are available at DDBJ/ENA/GenBank under BioProjectPRJNA702635

## Figures and Tables

**Table 1:** Chlorophyll content and number of nodules in *A. hypogaea* inoculated with *B. vignae* ISRA400 compared to not inoculated plants.

Treatments	Chlorophyll content (SPAD)	Number of nodules per plant (NN)	
Control NI	14.4b		0.5b
*B. vignae* ISRA400	37.9a		98a
			

Values followed by the same lower-case letters are not significantly different at the 5% level according to the Newman and Keuls test. Control NI: non-inoculated condition
